# IKULDAS: An Improved *k*NN-Based UHF RFID Indoor Localization Algorithm for Directional Radiation Scenario

**DOI:** 10.3390/s19040968

**Published:** 2019-02-25

**Authors:** Weiguang Shi, Jiangxia Du, Xiaowei Cao, Yang Yu, Yu Cao, Shuxia Yan, Chunya Ni

**Affiliations:** 1 School of Electronics and Information Engineering, Tianjin Polytechnic University, Tianjin 300387, China; shiweiguang@tjpu.edu.cn (W.S.); fri_ix@126.com (J.D.); tjpucxw@163.com (X.C.); 2 Tianjin Key Laboratory of Optoelectronic Detection Technology and Systems, Tianjin Polytechnic University, Tianjin 300387, China; 3 Department of Electrical and Electronic Engineering, Southern University of Science and Technology, Shenzhen 518055, China; issacyu@live.cn; 4 School of Marine Science and Technology, Tianjin University, Tianjin 300072, China; caoyu@tju.edu.cn; 5 China Mobile Communication Group Tianjin Co., Ltd., Tianjin 300308, China; nichunya@tj.chinamobile.com

**Keywords:** UHF RFID, directional radiation, indoor localization, *k*NN

## Abstract

Ultra high frequency radio frequency identification (UHF RFID)-based indoor localization technology has been a competitive candidate for context-awareness services. Previous works mainly utilize a simplified Friis transmission equation for simulating/rectifying received signal strength indicator (RSSI) values, in which the directional radiation of tag antenna and reader antenna was not fully considered, leading to unfavorable performance degradation. Moreover, a *k*-nearest neighbor (*k*NN) algorithm is widely used in existing systems, whereas the selection of an appropriate *k* value remains a critical issue. To solve such problems, this paper presents an improved *k*NN-based indoor localization algorithm for a directional radiation scenario, IKULDAS. Based on the gain features of dipole antenna and patch antenna, a novel RSSI estimation model is first established. By introducing the inclination angle and rotation angle to characterize the antenna postures, the gains of tag antenna and reader antenna referring to direct path and reflection paths are re-expressed. Then, three strategies are proposed and embedded into typical *k*NN for improving the localization performance. In IKULDAS, the optimal single fixed rotation angle is introduced for filtering a superior measurement and an NJW-based algorithm is advised for extracting nearest-neighbor reference tags. Furthermore, a dynamic mapping mechanism is proposed to accelerate the tracking process. Simulation results show that IKULDAS achieves a higher positioning accuracy and lower time consumption compared to other typical algorithms.

## 1. Introduction

With the proliferation of internet of things (IoT) technology, wireless indoor positioning has become an increasingly important issue and drawn plenty of attention in many fields, such as medical treatment [1], construction projects [2], supply chain [3], etc. Global position system (GPS), a sophisticated system, can be used to locate objects. However, it is not suitable for indoor localization because a direct line-of-sight (LOS) communication to the satellites required for GPS is not available in the indoor scene due to the obstruction of walls [4]. Many emerging technologies are exploited for indoor localization, including infrared [5], ultrasound [6], Bluetooth [7], wireless local area network (WLAN) [8], ultra-wide-band (UWB) [9], and radio frequency identification (RFID) [10]. Among them, RFID, especially UHF RFID, has become a promising alternative due to its non-line-of-sight, low complexity, compact size, and low cost [11,12,13,14]. Take the smartwatch and Bluetooth beacon necessary for [7] for instance, their price is generally over 7 dollars, whereas the price of a single passive UHF RFID tag is less than 5 cents.

Basically, there are two types of systems for UHF RFID indoor localization: range-based systems and range-free systems [15]. Range-based systems usually employ multiple RFID readers and use the geometric relationship between tags and readers (e.g., distance or angle) to calculate tags’ positions [11,12]. As for the range-free systems, capturing fine-grained fingerprints of the scenario, a great number of reference tags with determined locations are usually introduced to realize higher accuracy localization [13,14]. Since the cost of a tag is much lower than that of a reader, range-free based systems are universally more competitive, especially in the large-scale UHF RFID localization applications with multiple readers and multiple tags.

In range-free UHF RFID indoor localization systems, *k*-nearest-neighbor (*k*NN), a typical supervised learning algorithm, is widely used for meeting the demands of low computational cost and high scalability [13,16,17,18]. In *k*NN, the Euclidean distances in a measurement parameter (e.g., Received Signal Strength Indicator (RSSI) and radio frequency (RF) phase) between a tracking tag and all the reference tags are calculated, and the *k* reference tags with minimum distances are chosen as the nearest neighbors. By weighting the positions of nearest neighbors, the position of each tracking tag is obtained [13]. In general, the approaches utilizing the RF phase provide more accurate results. Typical examples include Tagoram [19], STPP [20], and TrackT [21]. Nevertheless, the reader reporting phase is much more expensive than those reporting RSSI. Impinj Speedway R420, the reader utilized by most RF phase-based approaches, is priced at over $1500. Whereas, Alien ALR-9680, similar product reporting RSSI information only, is priced lower than $600. Hence, our work is to offer a *k*NN-based approach with better performance and lower cost, by using a RSSI rather than RF phase.

During the simulation analysis and actual measurement, the Friis transmission equation (FTE) is commonly utilized for estimating the simulated RSSI values or rectifying the measured RSSI values. For ease of analysis, most prior research assumes that the tag antenna’s gain and the reader antenna’s gain in FTE are fixed values. However, in practical applications, patch antennas and dipole antennas are generally used as a commercial RFID reader antenna and tag antenna, respectively. According to the theoretical analysis and simulation data in [22], the gains of two above-mentioned antennas possess the characteristic of directional radiation. It means that the values of the gains are not fixed but depend on the radiation direction. Poor tag orientation with respect to the reader antennas would lead to an unfavorable RSSI distortion. Hence, most *k*NN-based UHF RFID localization systems cannot achieve the desired performance from such a simplified FTE when they are applied to a directional radiation scenario with patch antennas and dipole antennas. Some explorations have been made for an elaborate FTE [23,24]. In [23], by translating a modified gain model of patch reader antenna from a polar coordinate system to a Cartesian coordinate system, power estimation models for bistatic configuration and monostatic configuration were respectively proposed; however, they did not consider the effect of tag antenna’s gain model. In [24], a power estimation model was established by both considering and incorporating the gain models of patch antenna and dipole antenna into a Cartesian coordinate system. This model has a higher generality in practical situations. However, it is worth noting that the proposed power estimation models are deducted based on a premise that the posture (including azimuth and elevation) of the tag is fixed. Since the effect of tag’s posture on the gain directivity is not considered, the power estimation models in [24] cannot be directly applied to the scenarios of tags with complex and diverse postures. In addition, in [23,24], the channel path loss in the power estimation model is expressed on the basis of a simple LOS propagation where multipath propagation was not considered. More investigations are still needed for achieving a general and precise RSSI estimation model for a directional radiation scenario.

On the other hand, the value of *k* plays a vital role in the performance of *k*NN-based localization systems [25]. Once the environment factors (including topology of the reference tags and degree of the disturbance) change, the optimal value of *k* may fluctuate. A larger *k* does not mean more accurate but would introduce more computation. Some algorithms have been applied in *k*NN to determine an appropriate *k* [26,27]. In [26], an adaptive *k*NN algorithm is proposed. The *k* value contributing the best accuracy is acquired by pre-applying LANDMARC to the reference tag. In [27], *k*-means clustering divides all the tags into groups. Each tracking tag is assigned to a cluster with several reference tags. Considering that the reference tags in the same cluster share a high distance similarity with the tracking tag, they are chosen as the nearest neighbors, making the number of candidates more flexible. In recent years, some novel clustering algorithms with better performance have been developed, such as an expectation–maximization (EM) algorithm and spectral clustering algorithm [28]. However, to the best of our knowledge, applying such algorithms in *k*NN has not been reported in related works.

Besides accuracy, cost and time consumption are another two criteria for evaluating the performance of UHF RFID localization. Reducing the number of necessary reader antennas in the surveillance region is an effective solution for a lower cost. Inspired by the previous studies in which the radiation angle of the phased array antenna is adjusted to scan the surveillance region in turns [29], we plan to mount the reader antennas on a pan-tilt platform and make them scan in a rotation mode in this paper. In such a case, a surveillance region that originally requires multi fixed reader antennas can be covered by a rotating reader antenna. The rotation manner can be further well-designed to achieve a lower time consumption, according to the prior information such as the distribution of the tags.

In this paper, we proposed an improved *k*NN-based UHF RFID indoor localization algorithm for directional radiation scenarios, IKULDAS. With the facts mentioned above, we expect to achieve the localization performance of higher localization accuracy, lower cost, and less time consumption. The main contributions and novelties of this paper are as follows.

Firstly, the system is established by utilizing a reader antenna in a rotation mode. Compared with the conventionally fixed mode, the number of necessary reader antennas is reduced, saving the system cost.

Secondly, a novel RSSI estimation model for a directional radiation scenario is derived. Extended from our work in [24], the proposed model provides two-fold enhancement: (1) the effect of the tag’s posture on the gain directivity is considered; (2) the model is deducted on the basis of multipath propagation, in which the gain expressions of tag antenna and reader antenna for direct path and reflection paths are obtained.

Thirdly, three strategies are proposed for improving the *k*NN-based algorithm. The concept of optimal single fixed rotation angle (OSFRA) is introduced for extracting the RSSI measurements with superiority and the neighbor reference tags (NRT). An NJW-based clustering algorithm is designed for flexibly determining the nearest neighbor reference tags (NNRT). Compared with the clustering method in [24], the localization accuracy is improved. To accelerate the tracking process, a dynamic mapping mechanism (DMM) is also proposed, which enables the scanning mode of reader antenna to switch from coarse-grained scanning (CGS) to fine-grained scanning (FGS).

The outline of this paper is organized as follows. Section 2 surveys related works. In Section 3, the framework of the system under the directional radiation scenario is depicted and the corresponding RSSI estimation method is proposed. Section 4 presents the improved *k*NN-based algorithm with full implementation details. Simulation tests are carried out in Section 5, with results thoroughly analyzed. Section 6 provides the conclusion.

## 2. Related Work

For range-free UHF RFID indoor localization, quite a number of fingerprinting-based approaches have been proposed during the last decade. Fingerprinting-based approaches do not need information regarding the infrastructure and provide excellent performance. In [30,31], a mobile robot with RF antenna navigates in the monitoring region and records RSSI fingerprints at reference locations. By computing the similarities between current RFID measurements and recorded fingerprints, the tags can be tracked. The proliferation of multilayer perception algorithms such as artificial neural network and convolutional neural networks has offered a more competitive solution for training the recorded fingerprints. In [32], DeepFi incorporates a deep auto-encoder network for training subcarriers’ data over multiple antennas to extract the channel state information features for indoor localization. In [33], bi-modal data including subcarrier amplitude and phase is introduced to DeepFi for further improving the performance.

Despite their higher accuracy, such fingerprinting-based approaches need an offline stage for collecting fingerprinting information, which increases the time consumption and implementation complexity. Once the characteristics of the indoor environment change, the offline stage must be performed again. For proposing a positioning algorithm with low complexity and high universality, in this paper, we choose kNN employing reference tags and needing an online stage only as a research object and try to achieve targeted innovation.

## 3. System Setup and RSSI Estimation

**Figure 1:**
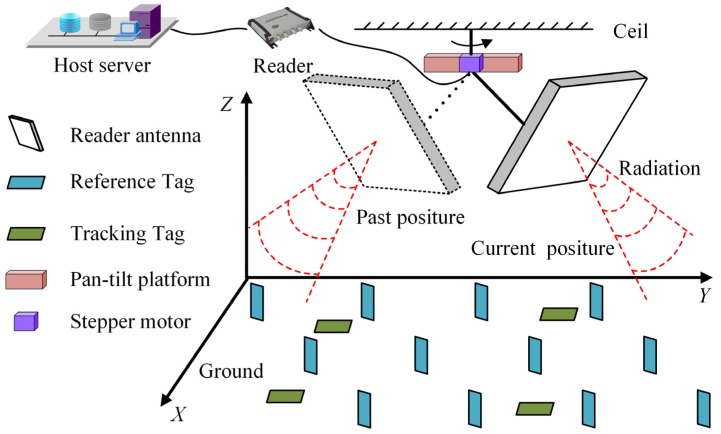
An illustrative diagram of the ultra high frequency radio frequency identification (UHF RFID) localization system performed under a directional radiation scenario.

As shown in Figure 1, we consider a UHF RFID localization system performed under a directional radiation scenario. Four main parts constitute the system: a reader with an antenna mounted on a pan-tilt platform, a certain number of passive tags being tracked, a certain number of reference tags with known locations, and a host server for running a positioning algorithm. In accordance with reality, the directional patch antenna and the dipole antenna are chosen as the reader antenna and the tag antenna, respectively. Compared with the omnidirectional reader antenna, the directional patch antenna has a higher effective isotropic radiated power (EIRP) with <90 degree beam width, which is beneficial to increase the maximum interrogations range and alleviate the interrogation collision. A dipole tag antenna ensures that the signals from multiple propagation directions can be utilized for activating the wake-up circuit on the tag chip.

Due to the narrow beam width, the reader antenna needs to constantly adjust the radiation direction for visiting all the tags in the surveillance region. To achieve this, a stepper motor is used to rotate the reader antenna around a fixed axis penetrating the plane of the pan-tilt platform through the centroid. After rotating a circle of a 2*π* radian (termed as a cycle), the reader antenna could identify all the tags and obtain the RSSI fluctuation profile of each tag referring to the rotation process. Here, the priority of our work is to precisely estimate each RSSI value on the fluctuation profile.

### 3.1. Estimation Frame Based on the FTE

For a passive UHF RFID system, uplink and downlink constitute the communication link. Uplink refers to the signal propagation from reader to tag, while the downlink refers to the signal propagation from tag to reader. Either the on-chip RSSI obtained by the tags via the uplink or the reader RSSI obtained by the readers via the downlink can be utilized for localization [34]. Compared with the on-chip RSSI, reader RSSI has a higher resolution. Hence, in our work, reader RSSI is employed as the observation parameter for positioning. Supposing that the uplink is identical to the downlink due to the monostatic configuration illustrated in Figure 1, and applying a modified FTE [24], the reader RSSI (dBm), can be expressed as:

(1) $$P_{R,T}=\{\begin{array}{cc} 10\log_{10}\left(\tau \mu_{T}\rho_{L}P_{Tx}{\left|G_{T}^{0}G_{R}^{0}L(d_{0})\right|}^{2}\right)+30+\psi & \mathrm{if}\;P_{R,T}>P_{thres}\\ P_{thres} & \mathrm{if}\;P_{R,T}\leq P_{thres} \end{array},$$

where $P_{thres}$ is the sensitivity threshold of the reader. τ is the modulation efficiency. $\mu_{T}$ is the power transfer efficiency. $\rho_{L}$ is the polarization loss factor. $P_{Tx}$ is the transmission power generated by the reader. $G_{T}^{0}$ and $G_{R}^{0}$ are the gains of the tag antenna and the reader antenna for the direct path. ψ stands for an interference following Gaussian distribution with zero mean and σ root-mean-square error. $L(d_{0})$ stands for the path loss which can be defined by:

(2) $$L(d_{0})={\left(\frac{\lambda }{4\pi \eta d_{0}}\right)}^{2}{\left|1+\sum_{i=1}^{N}\Gamma_{i}\frac{G_{T}^{i}}{G_{T}^{0}}\frac{G_{R}^{i}}{G_{R}^{0}}\frac{d_{0}}{d_{i}}e^{-j2\pi \left(d_{i}-d_{0}\right)/\lambda }\right|}^{2},$$

with

(3) $$\Gamma_{i}=\left(\cos\theta_{i}-q\sqrt{\varepsilon_{i}-\sin^{2}\theta_{i}}\right)/\left(\cos\theta +q\sqrt{\varepsilon_{i}-\sin^{2}\theta_{i}}\right),$$

where λ is the signal wavelength. $d_{0}$ is the direct distance between the tag and the reader antenna, $d_{i}$ is the length of the *i*th reflection path. $G_{T}^{i}$, $G_{R}^{i}$ are the gain of tag antenna and reader antenna for the *i*th reflection path. η is the path loss exponent. *N* is the number of reflection paths. $\Gamma_{i}$ is the complex reflection coefficient for the *i*th reflection path. $\theta_{i}$ is the incident angle to the normal to the reflector and *q* is a polarization dependent factor. $\varepsilon_{i}$ is the complex permittivity of the reflector.

### 3.2. Fundamental Gain Features of Typical Antennas

**Figure 2:**
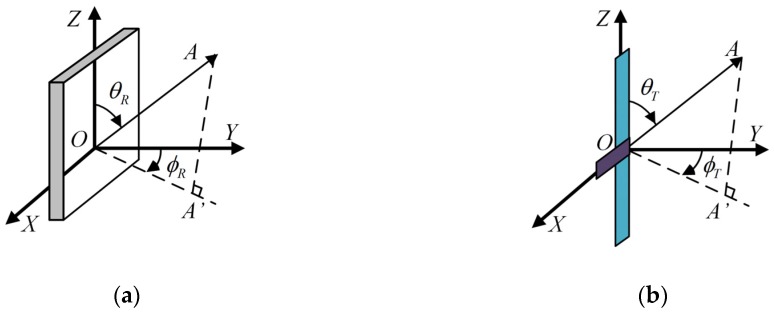
Spherical coordinate of discrete antenna. (**a**) Reader antenna with half-wave square patch. (**b**) Passive tag antenna with half-wave dipole.

As shown in (1) and (2), the gain features of the reader antenna and the tag antenna directly impact the precision of reader RSSI. Thus, achieving accurate expressions of the reader antenna’s gain and tag antenna’s gain carries critical importance for the RSSI estimation. Assuming that the size of the patch antenna satisfies the requirement of half-wavelength, and establishing a radiation scenario illustrated in Figure 2a, a modified gain of the ideal patch antenna can be obtained on the basis of the expression provided in [22].

(4) $$G_{R}(\theta_{R},\phi_{R})=3.136{\left[\tan(\theta_{R})\sin(0.5\pi \cos(\theta_{R}))\cos(0.5\pi \sin(\theta_{R})\sin(\phi_{R}))\right]}^{2},$$

where the surface of the reader antenna lies on the *XOZ* plane, and the centroid is at O, the pole of the coordinates system. Vector $\vec{OA}$ indicates the radiation direction of a certain beam of electromagnetic waves. $\theta_{R}$ is the angle from $\vec{OZ}$ to $\vec{OA}$. $\phi_{R}$ is the angle from $\vec{OY}$ to the projection of $\vec{OA}$ on the *XOY* plane. Similarly, establishing the radiation scenario illustrated in Figure 2b, the gain of a half-wavelength dipole tag antenna can be expressed as

(5) $$G_{T}(\theta_{T},\phi_{T})=1.641[\cos^{2}(0.5\pi \cos(\theta_{T}))]\sin^{-2}(\theta_{T}),$$

where the long side of tag antenna lies on the *Z*-axis, the short side of tag antenna lies on the *X*-axis. $\theta_{T}$ is the angle from $\vec{OZ}$ to $\vec{OA}$. $\phi_{T}$ is the angle from $\vec{OY}$ to the projection of $\vec{OA}$ on the *XOY* plane.

### 3.3. Gain Expressions Based on Position and Posture

**Figure 3:**
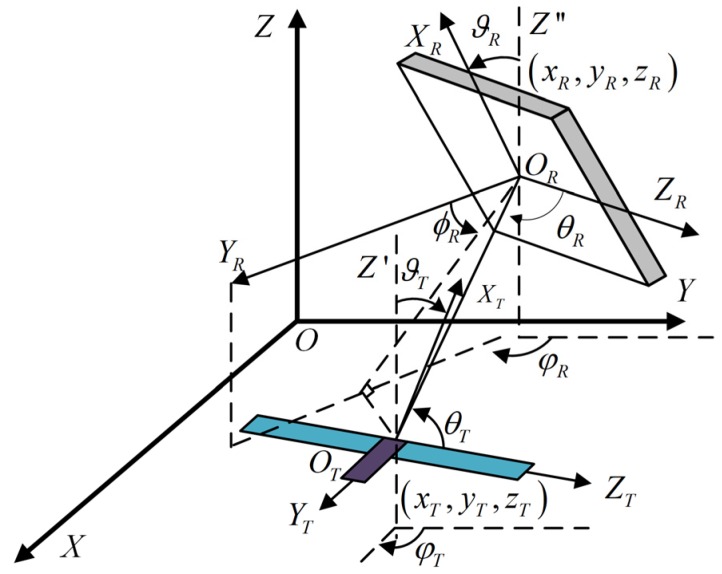
The Cartesian coordinate system for characterization of tag antenna and reader antenna.

For the UHF RFID system under a directional radiation scenario, the positions of tag antenna and reader antenna outline the transmission path (including a direct path and several reflection paths), while the postures of tag antenna and reader antenna determine the gain directivity. In this subsection, our goal is to express the gain features in (1) in terms of the positions and postures of tag antenna and reader antenna, by reformulating their gain models in Figure 2 into the same Cartesian coordinate system as that shown in Figure 3.

It is noteworthy that, though similar research has been done by us in [24], this paper provides a two-fold extension. Firstly, in Figure 3 of [24], for ease of analysis, we assumed that the surface where the passive tag lies is parallel to the *XOY* plane and the long side of the tag antenna is parallel to the *Y*-axis. Deducted from the premise that the posture of the tag is fixed, Equations (5) and (11) in [24] are not suitable for the scenarios with complex, diverse tags’ postures. To overcome this limitation, we take a further step to characterize the posture of tag antenna by using the inclination angle and rotation angle. Secondly, in [24], line-of-sight (LOS) communication is considered for ease of analysis, in which $L(d_{0})$ is simplified as $\lambda^{2}/{\left(4\pi \eta d_{0}\right)}^{2}$, and the approach for expressing $G_{T}^{i}$ and $G_{R}^{i}$ was not referred. In this work, we consider multipath communication depicted in (2) and elaborate a method for determining the expression of $G_{T}^{i}$ and $G_{R}^{i}$.

Suppose that the height of the reader antenna is much higher than that of the tag antennas. Also, the reader antenna radiates downward, as shown in Figure 3. The spatial relationship between the tag antenna and the reader antenna is described as follows. $\left(x_{T},y_{T},z_{T}\right)$ and $\left(x_{R},y_{R},z_{R}\right)$ denote the coordinates of the tag antenna and the reader antenna. $\left(\vartheta_{R},\varphi_{R}\right)$ and $\left(\vartheta_{T},\varphi_{T}\right)$ denote the postures of the tag antenna and the reader antenna, where $\vartheta_{R}$ is the inclination angle from $\vec{O_{R}X_{R}}$ to $\vec{O_{R}Z^{\prime \prime }}$, $\varphi_{R}$ is the rotation angle from $\vec{OY}$ to the projection of $\vec{O_{R}Y_{R}}$ on the *XOY* plane, $\vartheta_{T}$ is the inclination angle from $\vec{O_{T}X_{T}}$ to $\vec{O_{T}Z^{\prime }}$, $\varphi_{T}$ is the rotation angle from $\vec{OY}$ to the projection of $\vec{O_{T}Y_{T}}$ on the *XOY* plane. $\vec{O_{R}Z^{\prime \prime }}∥\vec{O_{T}Z^{\prime }}∥\vec{OZ}$.

Firstly, we choose $G_{T}^{0}$ and $G_{R}^{0}$ referring to the direct path as the analysis object. In such a case, the radiation direction $\vec{O_{T}O_{A}}$ is determined by the positions of the tag antenna and the reader antenna. Rotating and translating the coordinate system, then $\theta_{R}$, $\phi_{R}$, and $\theta_{T}$ can be expressed as:

(6) $$\theta_{R}=\arccos\left(Y_{1}/d\right),$$

(7) $$\phi_{R}=0.5\pi -\left[\arctan\left(X/Z\right)+\vartheta_{R}\right],$$

(8) $$\theta_{T}=\arccos\left(\left(Y_{2}\cos\left(\vartheta_{T}\right)+Z\sin\left(\vartheta_{T}\right)\right)/d\right),$$

with

(9) $$\left[\begin{array}{c} X\\ Y_{1}\\ Y_{2}\\ Z \end{array}\right]=\left[\begin{array}{ccc} \sin\varphi_{R} & \cos\varphi_{R} & 0\\ \cos\varphi_{R} & -\sin\varphi_{R} & 0\\ \cos\varphi_{T} & -\sin\varphi_{T} & 0\\ 0 & 0 & 1 \end{array}\right]\left[\begin{array}{c} x_{R,T}\\ y_{R,T}\\ z_{R,T} \end{array}\right],$$

where $d=\sqrt{{\left(x_{R,T}\right)}^{2}+{\left(y_{R,T}\right)}^{2}+{\left(z_{R,T}\right)}^{2}}$, $x_{R,T}=x_{R}-x_{T}$, $y_{R,T}=y_{R}-y_{T}$, $z_{R,T}=z_{R}-z_{T}$. Inserting (6) and (7) into (4), and (8) into (5), the expression of $G_{T}^{0}$ and $G_{R}^{0}$ can be obtained in terms of $\left(x_{R,T},y_{R,T},z_{R,T},\vartheta_{T}\right)$ and $\left(x_{R,T},y_{R,T},z_{R,T},\varphi_{R},\vartheta_{R}\right)$, respectively.

Secondly, our objective is to obtain the expression of $G_{T}^{i}$ and $G_{R}^{i}$ for each reflection path. In this work, a two-path propagation is considered. The reflection path only includes the path from the floor rather than those from the ceil, the obstacles, and the human bodies, due to the downward radiation of reader antenna and the convenience for analysis. By utilizing the reflection principle, the coordinate of the reflection point on the floor $\left(x_{r},y_{r},z_{r}\right)$ can be given by:

(10) $$\left(x_{r},y_{r},z_{r}\right)=\left(x_{R}-\frac{x_{R,T}z_{R}}{z_{T}{+z}_{R}},y_{R}-\frac{y_{R,T}z_{R}}{z_{T}{+z}_{R}},0\right),$$

For the uplink/downlink of the reflection path, the expression of $G_{R}^{i}$ can be obtained, by regarding the reflection point as the tag antenna (i.e., substituting $\left(x_{T},y_{T},z_{T}\right)$ by $\left(x_{r},y_{r},z_{r}\right)$) and recalculating (6), (7), and (4). Similarly, substituting $\left(x_{R},y_{R},z_{R}\right)$ by $\left(x_{r},y_{r},z_{r}\right)$ and recalculating (8) and (5), the expression of $G_{T}^{i}$ can be acquired.

## 4. Improved *k*NN-Based Algorithm

### 4.1. Selection of RSSI Measurement and NRT Based on OSFRA

**Figure 4:**
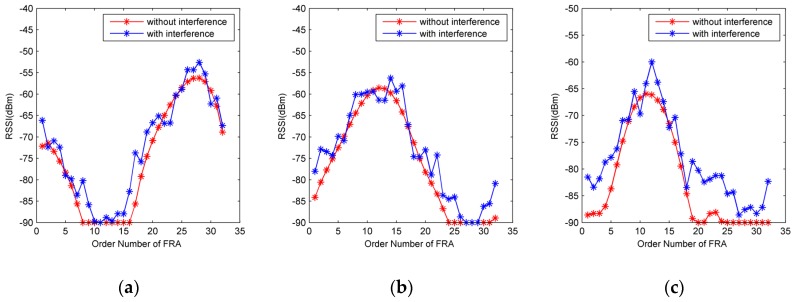
Received signal strength indicator (RSSI) profiles of three tags with σ = 20, $P_{Tx}$ = 2W. (**a**) tag 1, (**b**) tag 2, (**c**) tag 3.

By adopting the estimation method described in Section 2 and rotating the reader antenna for a cycle, a set of RSSI values can be obtained, forming a spatial-temporal RSSI fluctuation profile, as illustrated in Figure 4. Ideally, each point in the profile can be used for positioning. However, due to the interference ψ, a certain extent of degradation in accuracy would be caused, especially when such RSSI values are arbitrarily utilized without deliberation. In this subsection, we describe a method for selecting the superior RSSI measurements and NRT based on OSFRA. By doing this, a better positioning accuracy can be expected.

Firstly, a vector of fixed rotation angles (FRA) $\varphi_{F}=\{\varphi_{F}^{1},\varphi_{F}^{2},..,\varphi_{F}^{U}\}$ in ascending order are pre-designed for $\varphi_{R}$. *U* denotes the number of angles. Suppose the interval between two arbitrary adjacent FRA in $\varphi_{F}$ is the same, then we have $\varphi_{F}=\{\varphi_{F}^{1},\varphi_{F}^{1}+\varphi_{\mathrm{int}}^{},..,\varphi_{F}^{1}+\left(U-1\right)\varphi_{\mathrm{int}}^{}\}$. $\varphi_{\mathrm{int}}^{}$ denotes the interval. During the rotation of the reader antenna, the reader does not constantly contact with the tags and measure their RSSIs, but operates by stages. Only when $\varphi_{R}$ equals $\varphi_{F}^{u}$, the reader pauses the rotation and starts the scanning, u∈[1,U]. Two reasons account for this operation. On the one hand, most commercial off-the-shelf (COTS) readers estimate the RSSI by reducing the transmitting power step-by-step. A bigger range of power levels ensures a higher accuracy but costs more time on level switching. If the rotation speed is fast, it is hard to assign an appropriate rotation angle for each RSSI measurement. On the other hand, in the case with tags densely placed, the difference between the time stamp corresponding to the readers’ request and tag’s response cannot be predicted and neglected due to the reading collision. This difference would worsen the choosing of the appropriate rotation angle. During the scanning, for reducing the influence caused by ψ, we let the reader antenna remain stationary until the RSSI of each tag has been recorded *n* times. Such operation originates from equilibrium strategy. For a tracking tag, a vector of RSSI values with respect to $\varphi_{F}$ are depicted as $s_{T}=\{s_{T}^{\varphi_{F}^{1}},s_{T}^{\varphi_{F}^{2}},..,s_{T}^{\varphi_{F}^{U}}\}$ where $s_{T}^{\varphi_{F}^{u}}$ stands for the RSSI averaged by *n* measurements corresponding to $\varphi_{F}^{u}$.

Secondly, OSFRA is introduced for selecting a superior RSSI measurement and NRT. As shown in Figure 4, due to the prominent radiation directivity of the reader antenna and equilibrium strategy, the trend of RSSI profiles with interference basically agree with those without interference. Here, the fixed rotation angle possessing the maximum of actual $s_{T}$ is defined as OSFRA, given by:

(11) $$\varphi_{F}^{O}=\arg\underset{u}{\max}(s_{T}),$$

Suffering an interference following Gauss distribution, the average RSSI measurement achieved on OSFRA has a stronger interference immunity because of its bigger theoretical value, compared with the measurements obtained on the other FRA. Accordingly, it is reasonable to consider such an RSSI measurement obtained on OSFRA enjoying higher superiority. By a similar method, the OSFRA for each reference tag can be obtained. In our algorithm, for each tracking tag, we define the reference tags sharing the same OSFRA with it as NRT and only use their RSSI measurements for position estimation, rather than the RSSI measurements of other reference tags.

### 4.2. Determination of Nearest Neighbor Reference Tags Based on NJW

**Figure 5:**
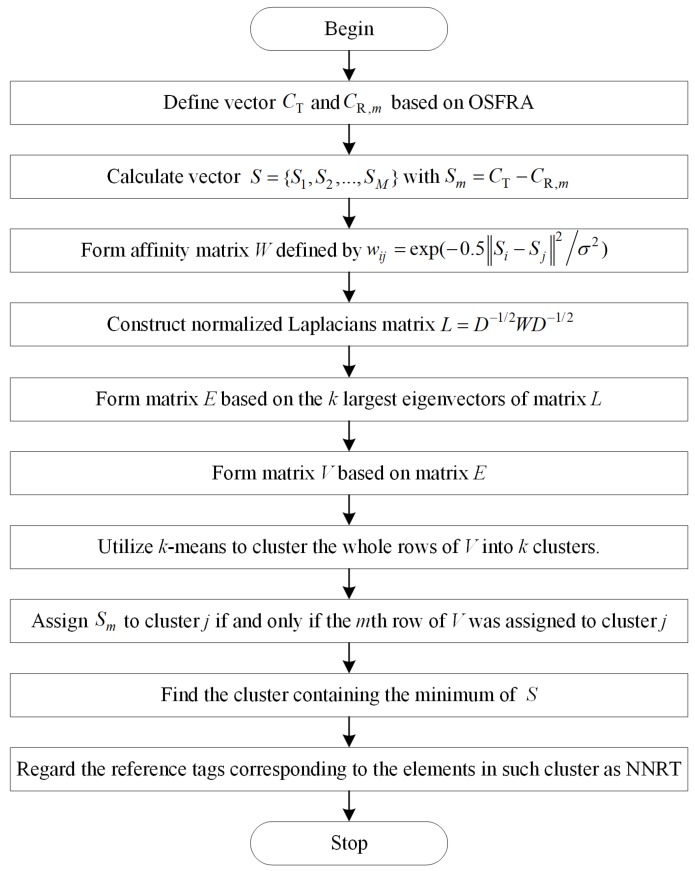
Flow chart of the NJW-based algorithm.

Instead of directly utilizing the measurements of total NRT for positioning, we propose an NJW-based algorithm to extract NNRT among NRT for further improving the accuracy. Inspired by the graphic partitioning theory, NJW aims to partition the observations into several clusters by constructing an affinity matrix based on Gaussian kernel function. Compared with *k*-means clustering, NJW has a better performance in the case of nonconvex distribution [28]. The procedure of our NJW-based algorithm shown in Figure 5 can be depicted as follows:

**Step 1**: OSFRA and two rotation angles adjacent to it are utilized to define the measurement vector. For the tracking tag, the measurement vector is $C_{T}=\{s_{T}^{\varphi_{F}^{O}-\varphi_{\mathrm{int}}^{}},s_{T}^{\varphi_{F}^{O}},s_{T}^{\varphi_{F}^{O}+\varphi_{\mathrm{int}}^{}}\}$. For the *m*th NRT, the measurement vector is $C_{R,m}=\{s_{R,m}^{\varphi_{F}^{O}-\varphi_{\mathrm{int}}^{}},s_{R,m}^{\varphi_{F}^{O}},s_{R,m}^{\varphi_{F}^{O}+\varphi_{\mathrm{int}}^{}}\}$. m∈[1,M]. *M* is the number of NRT. The reason why we do not simply use $s_{T}^{\varphi_{F}^{O}}$ and $s_{R,m}^{\varphi_{F}^{O}}$ as measurement vectors is due to the consideration of the symmetric ambiguity problem.

**Figure 6:**
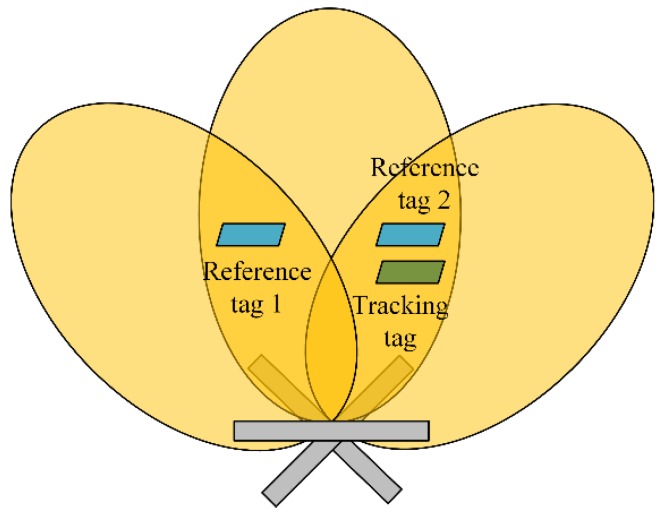
Schematic diagram of the symmetric ambiguity problem.

As shown in Figure 6, the shadow region in the middle represents the effective radiation area of OSFRA, and the shadow regions on both sides represent those of the adjacent rotation angles. Reference tag 1, Reference tag 2, and the tracking tag share the OSFRA. Also, the position of Reference tag 1 and Reference tag 2 are symmetrical within the middle shadow region. Here, the symmetric ambiguity problem can be depicted as follows. Since the two reference tags have the same theoretical RSSI value on $\varphi_{F}^{O}$, it is hard to decide which reference tag is a nearer NRT for the tracking tag by computing the distance between $C_{T}$ and $C_{R,m}$, when we only use $s_{T}^{\varphi_{F}^{O}}$, $s_{R,1}^{\varphi_{F}^{O}}$, and $s_{R,2}^{\varphi_{F}^{O}}$ to formulate measurement vectors. Undoubtedly, the introduction of $\varphi_{F}^{O}-\varphi_{\mathrm{int}}^{}$ and $\varphi_{F}^{O}+\varphi_{\mathrm{int}}^{}$ solves such a problem, since the two reference tags do not have the same theoretical RSSI values on $\varphi_{F}^{O}-\varphi_{\mathrm{int}}^{}$ and $\varphi_{F}^{O}+\varphi_{\mathrm{int}}^{}$ any longer.

**Step 2:** Define a relative measurement vector $S=\{S_{1},S_{2},\ldots ,S_{M}\}$ for clustering, with $S_{m}=C_{T}-C_{R,m}$. Then, form an M×M affinity matrix *W* in term of *S,* defined by $w_{ij}=\exp(-0.5{\left\|S_{i}-S_{j}\right\|}^{2}/\sigma^{2})$ if i≠j, and $w_{ii}=0$. σ denotes a scale factor determining the attenuation velocity. (1≤i≤M, 1≤j≤M). Then, calculate the diagonal matrix *D* defined by $D_{ii}=\sum_{n=1}^{M}w_{in}$ and $D_{ij}=0$ if i≠j, and construct the normalized Laplacians matrix $L=D^{-1/2}WD^{-1/2}$.

**Step 3:** Obtain $\alpha_{1},\alpha_{2}，\ldots ,\alpha_{k}$, the *k* largest eigenvectors of *L*, and form the matrix $E=\left[\alpha_{1},\alpha_{2},\ldots ,\alpha_{k}\right]$ by stacking the eigenvectors in columns. Further, form the matrix *V* based on *E* where $V_{ij}=E_{ij}{\left(\sum_{j=1}^{M}E_{ij}{}^{2}\right)}^{-1/2}$ and utilize the *k*-means algorithm to cluster the whole rows of *V* into *k* clusters. Finally, assign $S_{m}$ to cluster *j* if and only if the *m*th row of *V* was assigned to cluster *j*. For the *k*-means algorithm, the distances between each cluster centroid and the observations are calculated by cosine similarity, similar to the method in [27].

**Step 4:** Find the cluster containing the minimum of S and regard the reference tags corresponding to the elements in such cluster as the NNRT of the tracking tag.

As the NNRT have been decided, classical *k*NN is utilized to estimate the position of the tracking tag, given by $(x,y)=\sum_{h=1}^{H}w_{h}(x_{R,h},y_{R,h})$ with $w_{h}={\left(S_{N,h}\right)}^{-2}/\sum_{h=1}^{H}{\left(S_{N,h}\right)}^{-2}$. $\left(x_{R,h},y_{R,h}\right)$ is the coordinate of the *h*th NNRT. $S_{N,h}=\left|s_{T}^{\varphi_{F}^{O}}-s_{R,N,h}^{\varphi_{F}^{O}}\right|$ where $s_{R,N,h}^{\varphi_{F}^{O}}$ is the average RSSI measurement of the *h*th NNRT achieved on OSFRA. H is the number of NNRT referring to the tracking tag assigned by our NJW-based method.

It should also be noted that, besides the clustering methods, the clustering observations in [24] and our work are quite different. Instead of directly partitioning $S^{\prime }=\{C_{T},C_{R,1},C_{R,2},C_{R,3},\ldots C_{R,M}\}$ into *k* clusters, we choose S in the form of relative RSSI as the clustering objects, as depicted in Step 2. As the size of clustering objects has fallen by one, our method performs at a lower computing complexity.

### 4.3. Acceleration of Tracking Based on DMM

**Figure 7:**
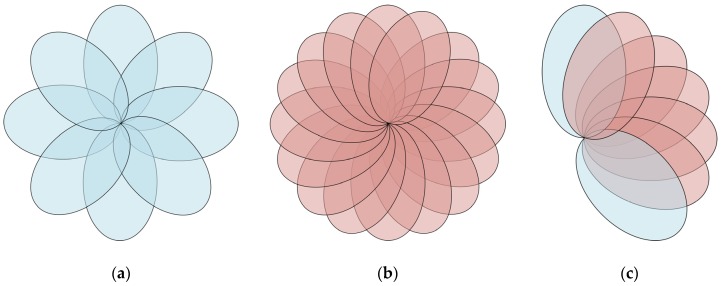
Scanning mode. (**a**) coarse-grained scanning (CGS) mode. (**b**) fine-grained scanning (FGS) mode. (**c**) Mapping from CGS to FGS.

As mentioned above, a set of FRA is pre-assigned for managing the scanning of the reader antenna. For ease of comparison and statement, we define the assignment with *U* no more than eight as CGS mode, and the assignment with *U* greater than 8 as FGS mode. It is conceivable that, compared with CGS, FGS would possess a higher accuracy, since a set of NRT with more pertinence would be obtained due to the smaller interval, as shown in Figure 7. However, the associated additional time consumption should not be neglected. Without considering the time consumed on algorithm running, the total time consumption for accomplishing one-time tracking, *T*, mainly includes two parts, given by:

(12) $$T=T_{R}+T_{S},$$

where $T_{R}$ denotes rotation time, $T_{S}$ denotes the scanning time.

For $T_{R}$, we have $T_{R}=2\pi /\omega$. ω represents the average rotation speeds. Suppose the average rotation speeds referring to coarse-grained scanning and fine-grained scanning are the same, the difference of $T_{R}$ between such two modes can be ignored.

For $T_{S}$, it can be depicted by $T_{S}=\sum_{u=1}^{U}\left(T_{S,u}\right)$ with $T_{S,u}=t\left(B_{u},n\right)$, where $T_{S,u}$ denotes the scanning time referring to $\varphi_{F}^{u}$ and is determined by $t\left(B_{u},n\right)$, a time consumption function for reading tags’ ID and measuring tags’ RSSI. $B_{u}$ denotes the number of tags detected by $\varphi_{F}^{u}$. In this paper, considering that most commercial readers report tags’ ID and tags’ RSSI synchronously with the help of a sophisticated anti-collision algorithm, we use a common anti-collision algorithm, the dynamic framed slotted ALOHA (DFSA) algorithm, to calculate $t\left(B_{u},n\right)$ [35].

Obviously, the value of *U* plays a great part in $T_{S}$. The difference in $T_{S}$ between the two modes grows as the gap on *U* increases. Especially, in the case with a tracking tag distributed mainly in a certain region for the fine-grained scanning mode, scanning spent on the FRA in which no OSFRA exists would be unnecessary and wasteful for $T_{S}$. Such waste influences the efficiency of tracking.

Aiming to solve the problem mentioned above, we present a mechanism, termed as DMM, for dynamically switching from CGS to FGS, in order to accelerate the tracking process. The procedure of DMM consists of two steps as follows.

**Step 1:** The reader antenna completes one cycle under a pre-designed CGS mode. For each tracking tag, its OSFRA are recorded. Then a mapping space for the *i*th tracking tag can be defined as $\left[\varphi_{F,C}^{O}-\varphi_{\mathrm{int},C}^{},\varphi_{F,C}^{O}+\varphi_{\mathrm{int},C}^{}\right]$, where $\varphi_{F,C}^{O}$ is the OSFRA under CGS, $\varphi_{\mathrm{int},C}^{}$ is the interval between two adjacent FRA under CGS. Furthermore, a set of mapping FRA under FGS can be obtained, given by:

(13) $$\varphi_{F,M,i}=\{\varphi_{F,C}^{O}-\varphi_{\mathrm{int},C}^{},\varphi_{F,C}^{O}-\varphi_{\mathrm{int},C}^{}+\varphi_{\mathrm{int},F}^{},\varphi_{F,C}^{O}-\varphi_{\mathrm{int},C}^{}+2\varphi_{\mathrm{int},F}^{},\ldots ,\varphi_{F,C}^{O}-\varphi_{\mathrm{int},C}^{}+w\varphi_{\mathrm{int},F}^{}\},$$

with

(14) $$\begin{array}{l} \varphi_{F,C}^{O}-\varphi_{\mathrm{int},C}^{}+w\varphi_{\mathrm{int},F}^{}<\varphi_{F,C}^{O}+\varphi_{\mathrm{int},C}^{}\\ \varphi_{F,C}^{O}-\varphi_{\mathrm{int},C}^{}+\left(w+1\right)\varphi_{\mathrm{int},F}^{}>\varphi_{F,C}^{O}+\varphi_{\mathrm{int},C}^{} \end{array}$$

where $\varphi_{\mathrm{int},F}^{}$ is the interval between two adjacent FRA under FGS.

**Step 2:** The reader antenna completes one cycle under a customized FGS mode. In such a customized mode, the total mapping FRA for the system, $\varphi_{F,M}$, is obtained by combining each $\varphi_{F,M,i}$, given by $\varphi_{F,M}=\varphi_{F,M,1}\cup \varphi_{F,M,2}\cup \ldots \cup \varphi_{F,M,Q}$, where *Q* is the number of tracking tags. During the cycle, the reader antenna stops to scan the tags only when $\varphi_{R}$ is equal to either element of $\varphi_{F,M}$.

Accordingly, for ease of contrast, the total time consumption for DMM is defined as:

(15) $$T^{D}=\left(T^{C}+T^{F,C}\right)/2,$$

where $T^{C}$ is the time consumption referring to CGS mode in Step 1, $T^{F,C}$ is the time consumption referring to the customized mode in Step 2.

## 5. Simulation and Analysis

### 5.1. Simulations Setup

For verifying the performance of IKULDAS, simulation experiments are conducted. A set of 21 × 21 reference tags are evenly distributed in the form of a regular grid in a 10 × 10 × 3 m^3^ room, while a total of 32 tracking tags for testing are deployed in a random style. The intervals on the *X*-axis and *Y*-axis between two adjacent reference tags are the same, equaling 0.5 m. The height of all the tags is 0.2 m. For all of the reference tags, the inclination angle and the rotation angle are set to 0.5π and 0, respectively. For all of the tracking tags, the inclination angle and the rotation angle are set to 0 and 0.5π, respectively. A reader antenna located in the center of the room with a height of 2.5 m, transmits and receives a 915 MHz carrier signal from the tags and the reader. For the settings of the reader and reader antenna, we have $P_{Tx}$ = 2W, ${\left|\Gamma \right|}^{2}$ = 0.1, τ = 0.5, $\mu_{T}$ = 1, $\rho_{L}$ = 0.5, η = 1, $P_{thres}$ = −90 dBm, ω = π rad/s, $\vartheta_{R}=\pi /4$. The RSSI estimation method proposed in Section 2 is utilized to simulate the RSSI measurement.

Especially, in order to investigate the effectiveness of the three strategies proposed, several localization schemes are introduced and compared. These schemes are as follows:

1. OSFRA+*k*NN: It uses OSFRA to choose the RSSI measurement with superiority and NRT, and adopts *k*NN to estimate the positions of the tracking tags. The *k* values for *k*NN are set to 3, 4, and 5 for testing.
2. RSFRA+*k*NN: It is similar to OSFRA+*k*NN except that the RSSI measurement and NRT are selected by a random single FRA.
3. OSFRA+*k-*means: The procedure is similar to of that of OSFRA+*k*NN. Whereas, the *k*NN algorithm is replaced by *k*-means clustering. The *k* values in *k*-means are set to 3, 4, and 5 for testing.
4. OSFRA+NJW: NJW is leveraged to replace *k*-means algorithm in OSFRA+*k*-means.
5. OSFRA+NJW+FGS: Based on OSFRA+NJW, it operates the reader antenna in a FGS mode.
6. IKULDAS: It denotes the algorithm simultaneously utilizing the three strategies proposed.

For obtaining statistical results, all the algorithms are executed 300 times according to the Monte Carlo method. Four indicators are used as a basis for the performance of the algorithms. Root mean square error (RMSE) and single estimated error (SER) are utilized to evaluate the localization accuracy. The average total time consumption (ATTC) and single total time consumption (STTC) are utilized to evaluate the localization efficiency.

### 5.2. Result and Comparisons

#### 5.2.1. Effectiveness of OSFRA

**Figure 8:**
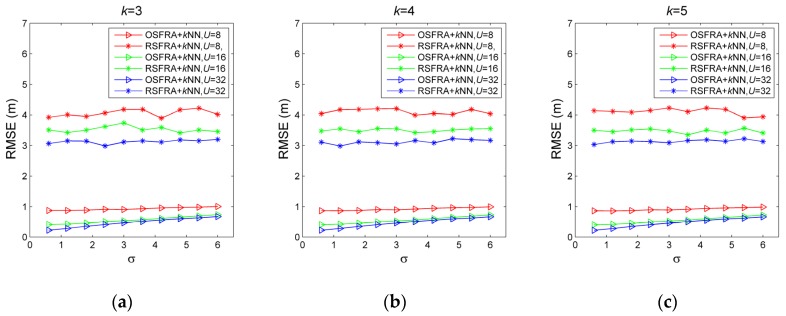
Root mean square error (RMSE) comparison for OSFRA+*k*NN and RSFRA+*k*NN under various σ, *U,* and *k*. (**a**) *k*=3, (**b**) *k*=4, and (**c**) *k*=5.

For verifying the effectiveness of OSFRA, the localization accuracy of OSFRA+*k*NN and RSFRA+*k*NN are compared. RSFRA, choosing the RSSI measurement with superiority and NRT by a random single FRA, is defined for comparison. *k*NN is utilized for position estimation. Figure 8 shows the RMSE comparison under various σ, *U,* and *k*. For one thing, for each set of σ, *U,* and *k,* it is obvious that OSFRA+*k*NN achieves a better performance, which proves the superiority of OSFRA. Take σ = 3, *U* = 16, and *k =* 5 for instance, the RMSE of OSFRA+*k*NN is 0.5261 m, while the corresponding value of RSFRA+*k*NN is 3.4748 m. For another, for the same set of *U* and *k*, the RMSE of OSFRA+*k*NN grows steadily as σ increases from 0.6 to 6, showing a good environmental adaptability. By contrast, the RMSE of RSFRA+*k*NN fluctuates greatly due to the uncertainty of the random FRA. Furthermore, for the same set of σ and *k*, the RMSE of OSFRA+*k*NN decreases as *U* increases from 8 to 32. Such increasing attributes to a set of NRT with more centralized deployment, benefiting from the shrink of the interval between two adjacent FRA from π/4 to π/16. Also, it is conceivable that such accuracy increases will become very small once *U* reaches a critical value.

#### 5.2.2. Effectiveness of NJW-Based Algorithm

**Figure 9:**
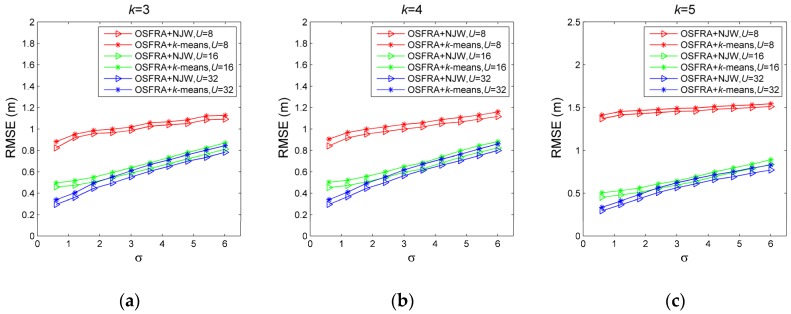
RMSE comparison for OSFRA+ NJW and OSFRA +*k-*means under various σ, *U,* and *k*. (**a**) *k* = 3, (**b**) *k* = 4, and (**c**) *k* = 5.

The localization accuracy of OSFRA+NJW and OSFRA+*k-*means are compared in this subsection to investigate the effectiveness of the NJW-based algorithm. As shown in Figure 9, for each set of σ, *U,* and *k,* the RMSE of OSFRA+NJW is always lower than that of OSFRA+*k-*means. Take σ = 1.2, *U* = 32, and *k =* 5 for instance, the RMSE of OSFRA+ NJW is 0.3674 m, while the corresponding value of OSFRA+*k-*means is 0.4101 m. In addition, the relative improvement of OSFRA+NJW referring to OSFRA+*k*-means, given by $I=\left(RMSE_{OSFRA+NJW}-RMSE_{OSFRA+k-means}\right)/RMSE_{OSFRA+k-means}$, is listed in Table 1, revealing the superiority of OSFRA+NJW in a more visible way. Compared with the cases with large σ, the relative improvement in the cases with small σ are more remarkable. Especially when *U* = 32 and σ≤1.2, the superiority exceeds 10%. As σ increases, the superiority decreases gradually but always exists. Even for the cases with σ = 6, the average relative improvement reaches 5.93%. Such behavior demonstrates the stability of OSFRA+NJW.

#### 5.2.3. Effectiveness of DMM

**Figure 10:**
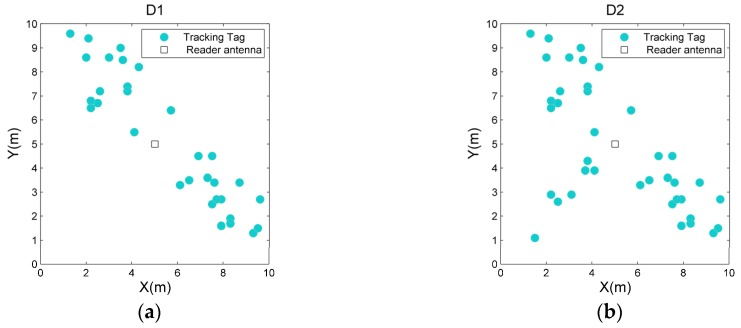
Diagram of two deployments with regard to the tracking tags. (**a**) D1 and (**b**) D2.

**Figure 11:**
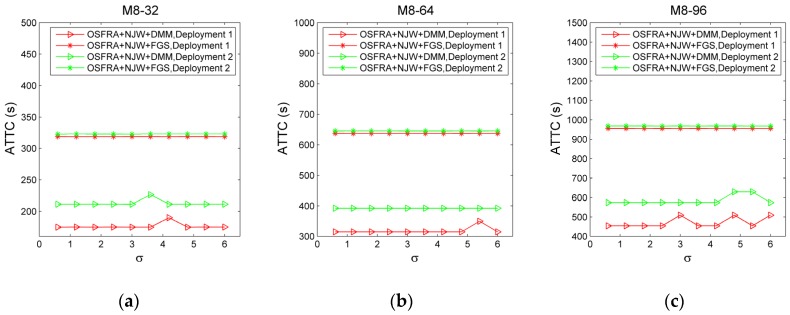
The average total time consumption (ATTC) comparison for OSFRA+NJW+DMM and OSFRA+NJW+FGS under various σ, mapping instances and tracking tag deployments. (**a**) M8-32. (**b**) M8-64. (**c**) M8-96.

Since the data in Figure 8, Figure 9, and Table 1 has revealed the advantages of FGS on the accuracy, we use ATTC as the basis to compare the performance of OSFRA+NJW+DMM and OSFRA+NJW+FGS for validating the effectiveness of DMM. Three mapping instances, namely M8-32, M8-64, and M8-96, are provided for comparison. For three such instances, $\varphi_{\mathrm{int},C}^{}$ in OSFRA+NJW+DMM equals 2π/8, $\varphi_{\mathrm{int},F}^{}$ in OSFRA+NJW+DMM equals 2π/32, 2π/64, and 2π/96, respectively. The setting of $\varphi_{\mathrm{int}}^{}$ in OSFRA+NJW+FGS is the same as those of $\varphi_{\mathrm{int},F}^{}$. Furthermore, two deployments with regard to the tracking tags, namely D1 and D2, are provided, among which the tracking tags in D1 are mainly distributed in two regions and the tracking tags in D2 are mainly distributed in three regions, as shown in Figure 10. Figure 11 shows the ATTC comparison for OSFRA+NJW+DMM and OSFRA+NJW+FGS. Evidently, OSFRA+NJW+DMM has a lower time consumption. Especially in D2, such an advantage is more outstanding. Note, the ATTC sensitivity of OSFRA+NJW+DMM with regard to σ is slightly higher than those of OSFRA+NJW+FGS, since the uncertainty of $\varphi_{F,M}$ becomes more serious when σ increases to a larger value. Still, the merit of OSFRA+NJW+DMM outweighs such a flaw. On the other hand, the improvement degree on ATTC averaged by σ rises as the angles number of FGS increases. Take D1 for instance, the improvement degree averaged by σ under M8-32 is 40.16%, whereas the corresponding value under M8-96 is 46.16%.

#### 5.2.4. Performance of IKULDAS Under Various Conditions

**Figure 12:**
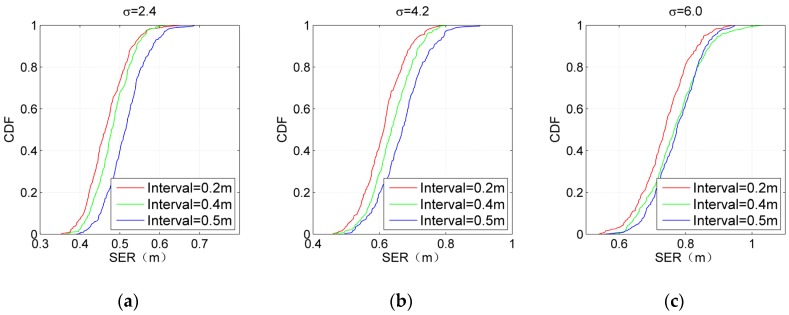
Single estimated error (SER) of IKULDAS under various reference tag densities and σ. (**a**) σ = 2.4. (**b**) σ = 4.2. (**c**) σ = 6.

**Figure 13:**
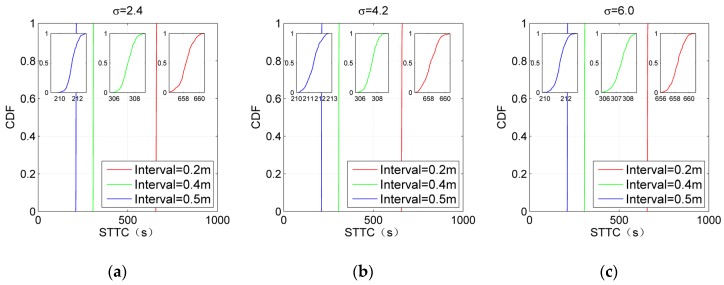
Single total time consumption (STTC) of IKULDAS under various reference tag densities and σ. (**a**) σ = 2.4. (**b**) σ = 4.2. (**c**) σ = 6.

For comprehensively evaluating the performance of IKULDAS, the interval between two adjacent reference tags is adjusted to be 0.2, 0.4, and 0.5, respectively. D1 and M8-32 are chosen as the scenario conditions. The number of clusters for NJW is set to four. Figure 12; Figure 13 depict the accuracy and efficiency in the form of the cumulative distribution function (CDF). In the case of Figure 12a, it can be seen that the 50th percentile of SER with 0.2, 0.4, and 0.5 interval are below 0.52 m, while the improvement from the 0.4 interval to the 0.2 interval is weaker than those from the 0.5 interval to the 0.4 interval. Such a gap in the improvement illustrates that the improvement resulting from tag density would reach an upper bound as long as the interval arrives at a critical value. Note that the feature of such a gap changes as σ increases. As shown in Figure 12c, the improvement from the 0.5 interval to the 0.4 interval blurs due to the large deviation of RSSI caused by larger σ, while the improvement from the 0.4 interval to the 0.2 interval is still clear. It can be concluded that a larger density of reference tags is essential for the accuracy in the high-noise cases. As for Figure 13, it can be seen that the decrease in the interval would aggravate the time consumption. Clearly, an appropriate interval can be obtained by carefully balancing the context requirement of the accuracy and time consumption.

## 6. Conclusions

In this paper, we present an improved *k*NN-based UHF RFID indoor localization algorithm for a directional radiation scenario. For reducing the quantity of necessary reader antennas for the surveillance region, our system employs a single reader antenna operating in a rotation mode. A precise RSSI estimation model is established based on the Friis transmission equation and the radiation of both patch antenna and dipole antenna. In this case, the influence of the tag’s posture on the gain directivity and multipath effect are fully considered. For enhancing the performance of *k*NN-based localization, IKULDAS is proposed, which is formed by three novel strategies incorporated into a typical *k*NN. Various mapping instances, deployments with regard to the tracking tags, and densities of the reference tags are provided to comprehensively evaluate the performance of the algorithm. Simulation results demonstrate the superiority of IKULDAS as it provides higher accuracy and lower time consumption than typical algorithms. In our work, the influence of the multipath effect only refers to the reflection of the floor whereas the obstacles such as the human body and furniture are not taken into account. More investigations are underway in this direction. The NJW clustering algorithm makes the number of NNRT for each tracking tag more flexible. However, how to choose an appropriate number of clusters according to the assignment of reference tags remains a problem. Our future work will focus on the intensive exploration of spectral clustering and the dynamic selection of the optimal number of clusters.

## Figures and Tables

**Table 1:** Relative improvement of OSFRA+NJW referring to OSFRA+*k*-means.

		k	*U* = 8			*U* = 16			*U* = 32		
	I										
σ			3	4	5	3	4	5	3	4	5
0.6			6.66%	7.69%	3.03%	7.69%	9.91%	10.25%	12.34%	12.45%	12.11%
1.2			3.38%	8.22%	2.64%	8.22%	9.28%	9.32%	10.29%	10.38%	10.29%
1.8			3.00%	7.95%	2.38%	7.95%	9.03%	9.16%	10.05%	9.52%	9.67%
2.4			3.33%	7.97%	2.32%	7.97%	8.64%	8.79%	9.96%	9.14%	9.78%
3.0			3.23%	7.73%	2.40%	7.73%	7.94%	7.69%	9.39%	8.80%	9.26%
3.6			3.21%	7.81%	2.37%	7.81%	8.34%	7.91%	9.17%	8.70%	8.80%
4.2			2.73%	7.81%	2.11%	7.81%	7.40%	7.17%	8.44%	8.18%	7.85%
4.8			3.25%	7.41%	2.21%	7.41%	7.47%	6.71%	7.78%	8.36%	7.84%
5.4			3.22%	7.13%	2.09%	7.13%	7.33%	6.18%	8.17%	8.27%	7.42%
6.0			3.11%	6.73%	2.10%	6.73%	6.57%	6.07%	7.25%	7.78%	7.11%
